# Eye Size Affects Cuteness in Different Facial Expressions and Ages

**DOI:** 10.3389/fpsyg.2021.674456

**Published:** 2022-01-11

**Authors:** Lichang Yao, Qi Dai, Qiong Wu, Yang Liu, Yiyang Yu, Ting Guo, Mengni Zhou, Jiajia Yang, Satoshi Takahashi, Yoshimichi Ejima, Jinglong Wu

**Affiliations:** ^1^Cognitive Neuroscience Laboratory, Graduate School of Interdisciplinary Science and Engineering in Health Systems, Okayama University, Okayama, Japan; ^2^School of Education, Suzhou University of Science and Technology, Suzhou, China; ^3^Cognitive Neuroscience Laboratory, Graduate School of Natural Science and Technology, Okayama University, Okayama, Japan; ^4^Research Center for Medical Artificial Intelligence, Shenzhen Institute of Advanced Technology, Chinese Academy of Science, Shenzhen, China; ^5^School of Mechatronical Engineering, Beijing Institute of Technology, Beijing, China

**Keywords:** cuteness, baby schema, eye size, facial expression, age

## Abstract

Researchers have suggested that infants exhibiting baby schema are considered cute. These similar studies have mainly focused on changes in overall baby schema facial features. However, whether a change in only eye size affects the perception of cuteness across different facial expressions and ages has not been explicitly evaluated until now. In the present study, a paired comparison method and 7-point scale were used to investigate the effects of eye size on perceived cuteness across facial expressions (positive, neutral, and negative) and ages (adults and infants). The results show that stimuli with large eyes were perceived to be cuter than both unmanipulated eyes and small eyes across all facial expressions and age groups. This suggests not only that the effect of baby schema on cuteness is based on changes in a set of features but also that eye size as an individual feature can affect the perception of cuteness.

## Introduction

The face is a special visual stimulus that can convey emotional information, such as a sense of beauty and cuteness, to others. Cuteness is a positive stimulus with biological significance that is often a feature of immature and vulnerable objects, such as infants, children, or young animals. Thus, it inspires the empathy and compassion of observers (Glocker et al., 2009a,b; Buckley, 2016; Yin et al., 2017). For example, some existing studies have shown that the perception of cuteness of children declines as children age (Luo and Li, 2011; Luo et al., 2015a; Yin et al., 2017), and adults think that children with positive expressions are cuter than children with neutral or negative expressions (Aradhye and Vonk, 2015). In addition, Konrad Lorenz defined the “Kindchenschema” as an innate releasing mechanism for caretaking behavior (Lorenz, 1943) and proposed baby schema as a set of infantile traits, such as a large head, a high and protruding forehead, large eyes, chubby cheeks, a small nose and mouth, short and thick extremities, and a plump body shape, which together trigger such a mechanism (Glocker et al., 2009a). Children with these features are considered cuter than others (Lorenz, 1943; Glocker et al., 2009a,b), and cuter facial features can attract attention and promote adult caretaking (Brosch and Sander, 2007; Luo et al., 2015b).

After Lorenz proposed the concept of a baby schema, Glocker et al. conducted a study taking the line of the inside corner of the eye as the abscissa and the midline of the nose bridge as the ordinate (Glocker et al., 2009a). The authors used the pixel value of the length of the head as the absolute metric and quantitatively changed all facial features mentioned in the baby schema, such as the length of the forehead and the size of the eyes, nose, and mouth (Glocker et al., 2009a). During this study, they further demonstrated that compared to infant faces with neutral or low baby schema, infant faces with high baby schema were considered to be cuter and stimulated stronger care motivation (Glocker et al., 2009a). A study by Borgi et al. found that the change in cuteness brought about by the baby schema is not limited to the faces of the infants, as adults with higher baby schema facial features are also considered to be cuter than adults without such features (Borgi et al., 2014). Dijker et al.'s work in 2017 verified that having physical baby schema features, such as a fattier body and a higher size ratio of the head to the body, make people appear like children (Dijker et al., 2017). Later, Almanza-Sepúlveda et al., in a 2018 study, quantified features and found that people felt that babies with a larger cephalic curvature, a smaller chin, and round chubby features were cuter (Almanza-Sepúlveda et al., 2018). The above research shows that facial features play an important role in the perception of cuteness.

Furthermore, in research on facial features, both overall features and individual features affect the perception of facial information. For example, in a study about the correction degree of nasal deviation and facial attractiveness, the researchers found that changes in the degree of correction can affect attractiveness (Bui et al., 2015). In Hedwig Eisenbarth et al.'s study, the authors found that faces with different expressions were not equally decoded. For example, compared with sad and fearful facial expressions, when happy expressions were observed, participants usually fixated on the mouth region longer (Eisenbarth, 2011). Ryan and Schwartz found that when participants were asked to select “the face that they saw,” the error rate of identifying a face based on the shape of the eyes was lower. In contrast, the size and spatial location of facial features (eyebrow shape, mouth shape, eye–eye distance, eye–nose distance, and nose–mouth distance) do not induce a significant change in the perceived emotion (Ryan, 2013). These results indicate that individual facial features may be more conducive to facial information perception than overall features are.

The eyes are an important facial feature. Emery argued that the eyes contain more important information about face identity and emotional state than other features do (Emery, 2000). Schyns et al. found that the main factor in determining the identity and gender of a face is visual information about the eyes (Schyns and Bonnar, 2002). Additionally, various studies have shown that the perception of eyes might be uniquely influential (Grossmann, 2017). The results of some previous studies have shown that eyes can attract attention or affect the attentional preferences of others. In a study conducted by Marta Borgi et al. in 2014, faces of infants were divided into three areas of interest (eyes, nose, and mouth), and the number of fixations and the fixation duration in each area were measured (Borgi et al., 2014). The results show that observers focused on the areas of the eyes rather than the areas of the nose and mouth (Borgi et al., 2014). Some studies have also shown that infants notice the eyes before recognizing the face (Taylor et al., 2001). However, eye size is an individual feature, and whether it affects the perception of cuteness on its own has not yet been clarified.

The aim of the present study was to investigate the effect of eye size on cuteness by the controlled manipulation of eye size in photos of people with different facial expressions and of different ages. A hypothesis was proposed that just changing the eye size, without changing overall facial features might affect the cuteness perception of the observer. To test this hypothesis, eye sizes with different facial expressions and ages were changed and categorized as unmanipulated, larger, or smaller than the original aspect ratio and pupil position. Two experiments were conducted to examine the eye sizes in different types of facial expressions (positive, neutral, and negative) and across different ages (adults and infants) to investigate how these aspects affect the perception of cuteness. In the first experiment, pair comparison methods were used to contrast different expressions and eye sizes of the same model to more clearly examine the effect of local features on cuteness. In experiment 2, the 7-point scoring method investigated whether eye sizes influence cuteness across ages and verified the previous research.

## Method

### Participants

Statistical power analysis in G^*^Power version 3.1 was performed for sample size estimation (Faul et al., 2007). The projected partial η^2^ of the interaction of this experiment was set at 0.25, the two-tailed alpha level was set at 0.05; the power value was set at 0.95, the number of groups was set at 1, and the number of measurements was set at 9 for experiment 1 and at 18 for experiment 2. Subsequently, it was determined that sample sizes of 22 for experiment 1 and 14 for experiment 2 were required. Therefore, we recruited 24 Asian students (25.42 ± 3.76 years old, 20 men and 4 women) from Okayama University with no previous reproductive history. Every participant reported their medical history and provided written informed consent to participate. This experiment was previously approved by the Ethics Committee of Okayama University and was conducted in accordance with the Declaration of Helsinki.

### Materials

#### Stimuli Selection

A total of 229 photos depicting a face without an exposed mouth or scars (127 photos of 20 adults and 20 children with various facial expressions) were chosen from the KDEF (Lundqvist et al., 1998), café (LoBue and Thrasher, 2015), and TIF (Maack et al., 2017) databases. Then, an oval with a height of 500 pixels and a width of 400 pixels was used to choose the face for each photo. All photos were set to grayscale and printed on A4 paper, with 6 photos per sheet. For the stimuli selection task, 10 Asian students (26.1 ± 2.96 years old, 5 men and 5 women did not coincide with the participants of the following two experiments) from Okayama University who had no previous reproductive history were chosen to participate. These 10 students evaluated each stimulus as follows:

(1) Perceived age: (0–36 years, divided into 8 groups of 4 years each).
(2) Skin color: (1: Light to 7: Dark).
(3) Degree of obesity: (1: Thin to 7: Fat).
(4) Eye size: (1: Small eyes to 7: Big eyes).
(5) Nose size: (1: Small nose to 7: Big nose).
(6) Mouth size: (1: Small mouth to 7: Big mouth).
(7) Sex: (1: Male, 2: Not sure, or 3: Female).
(8) Expression types: (1: Negative, 2: Neutral, or 3: Positive).

Based on the evaluation results of 10 participants, the score of each photo in each evaluation was calculated. Since the above scores are mainly an evaluation of facial features, and the facial features of adults and children are quite different due to the growth and development of the former, all the photos were divided into an adult group and an infant group according to the age rating. On the basis of the sex rating, photos in the adult group for which more than 70% of the participants indicated that the sex was unknown were deleted. Then, except for sex, the mean of each score and 2 SDs were calculated for each group, and photos with scores beyond 2 SDs were deleted. Finally, after the evaluation of the types of facial expressions, photos in which the facial expression was recognizable by more than 70% of the students were selected. All photos of the same model were deleted when there were fewer than three expression categories (positive, neutral, and negative). When there was more than one photo of the same model with the same expression type, the photo with a smaller facial angle was selected.

As a result, photos of five adult models and six infant models exhibiting three types of facial expressions were selected. To equalize the number of photos between the two age conditions, the models in the infant group whose actual age was more than 2 SDs from the mean were removed. Ultimately, a total of 30 photos of 5 adults (20–30 years old) and 5 infants (4–12 months old) covering 3 types of facial expressions (negative, neutral, and positive) for each model are chosen, as shown in Table 1. Based on the classification of image stimuli in the original database, the negative expressions of adults included sadness, and the negative expressions of the infants included disgust and sadness.

**Table 1:** The 30 selected stimuli.

**Adults**			**Infants**		
**Positive**	**Neutral**	**Negative**	**Positive**	**Neutral**	**Negative**
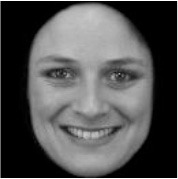	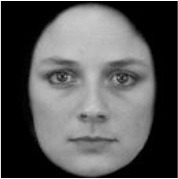	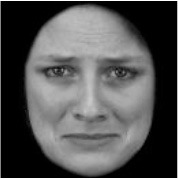	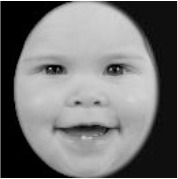	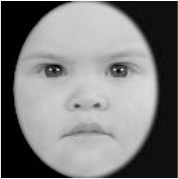	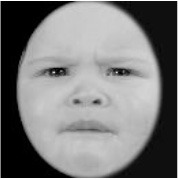
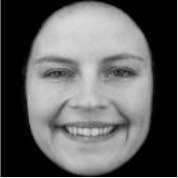	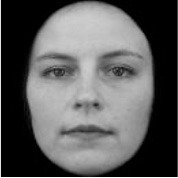	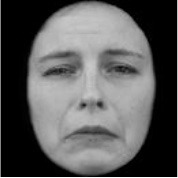	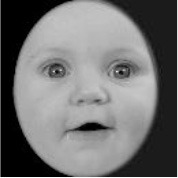	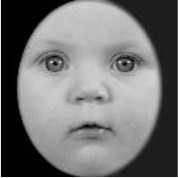	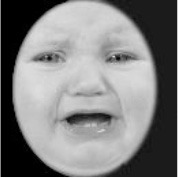
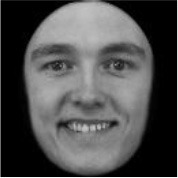	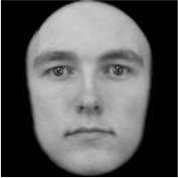	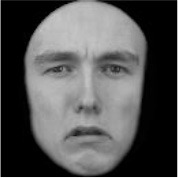	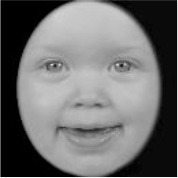	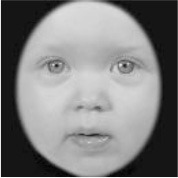	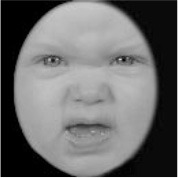
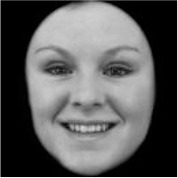	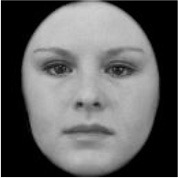	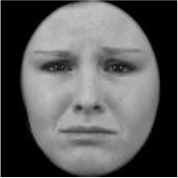	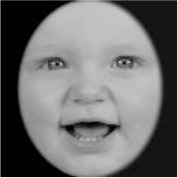	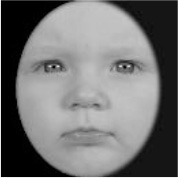	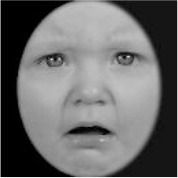
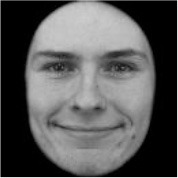	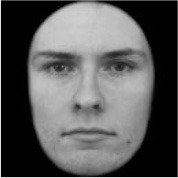	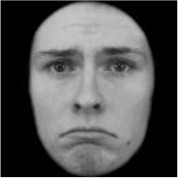	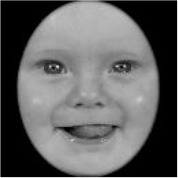	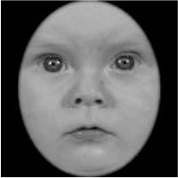	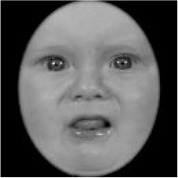

*Following the evaluation by the participants, 5 adult models and 5 infant models, each with 3 types of expressions, were selected for a total of 30 photos*.

#### Creative Stimuli Production

**Figure 1:**
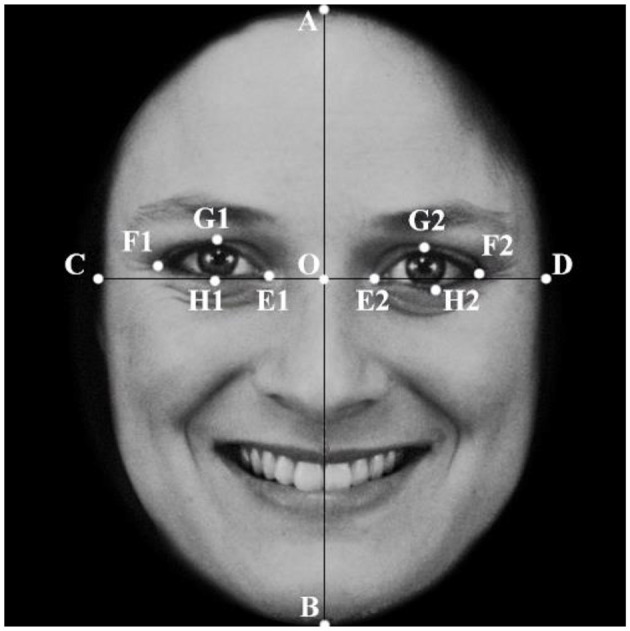
Face ratio measurement method. Using the facial measurement method, a coordinate system is established for the stimuli faces, using the line of the inside of the eye as the X-axis and the midline of the nose as the Y-axis. The intersection of the X-axis and the Y-axis (O), head length (AB), face width (CD), eye width (EF) and length (GH), eye aspect ratio (GH/EF), and eye-to-face length ratio (GH/EF) were evaluated.

To study the effect of changes in facial proportion due to changes in eye size on cuteness-related emotion, eye sizes were modified in the 30 selected facial photos. Using anthropometric methods consistent with those found in previous studies, a coordinate system was superimposed on the face in each photograph, with the X-axis connected to the inner angle of the eye and the Y-axis going across the midline of the nose. Facial data are obtained by measuring the distance between the following features, as shown in Figure 1: A (top of the head), B (bottom of the chin), C and D (outer edges of the face along the X-axis), E1 and E2 (inner corners of the eyes), F1 and F2 (outer corners of the eyes), G1 and G2 (upper edges of the eyes), H1 and H2 (the lower edge of the eye), and O (the base of the nose where the X- and Y-axes intersect).

The 30 pictures were divided into two groups (adults and infants). Each group was divided into 3 categories according to the type of expression. Then, the length of the face (AB, fixed value) and the width and length of the eye (EF and GH, respectively) were measured, and the eye aspect ratio (GH/EF) and eye-to-face length ratio (GH/EF) were calculated. The averages of the calculated results are shown in Table 2.

**Table 2:** Average calculated results.

**Stimulus types**	**Adults**			**Infants**		
**Expression features**	**Negative**	**Neutral**	**Positive**	**Negative**	**Neutral**	**Positive**
Head length (AB, fixed value)	500 pixels	500 pixels	500 pixels	500 pixels	500 pixels	500 pixels
Eye aspect ratio (GH/EF)	0.436	0.470	0.440	0.407	0.547	0.490
Eye-to-face length ratio (GH/AB)	0.085	0.091	0.086	0.091	0.117	0.010

A head height of 500 pixels was used as the reference, and EF/GH was used as the aspect ratio of the eyes. The length of the face and the aspect ratio of the eyes were maintained. Similar to previous studies, Photoshop (Adobe Systems, San Jose, CA, USA) was used to objectively quantify and parametrically enlarge or shrink the eye-to-face length ratio (GH/AB) around the center of the pupil to obtain eyes of different sizes (Glocker et al., 2009a). To make the change in the size of the eyes sufficiently obvious, various eye sizes were used. To ensure the effectiveness of this technique, an evaluation test was performed, and the eyes were determined to still look sufficiently natural when magnified by 15%. However, when eyes were enlarged by more than 15%, the lateral aspect (F) of the eyes of the infant exceeded the edge of the face (point C or D), and excessively enlarged eyes may obscure the eyebrows. Therefore, the maximum proportion of eye enlargement was set at 15%. Additionally, to make the eye variables consistent, the original eye ratio (EF/GH) of adults and infants remained unchanged when the eye-to-face length ratio (GH/AB) was increased or decreased by 15%.

All 30 photos were altered as described above. Table 3 shows example photos of an adult and an infant with a positive expression. The resulting images of faces with large eyes (+15% eye-to-face length ratio), medium eyes (unmanipulated eye-to-face length ratio), and small eyes (−15% eye-to-face length ratio) were then used for the experiment. That is, 9 photos of each model (90 photos in total) were used as experimental stimuli for this study.

**Table 3:** Example stimuli.

	**Small eyes (−15% Eye-to-face length ratio)**	**Medium eyes (Unmanipulated Eye-to-face length ratio)**	**Large eyes (+15% Eye-to-face length ratio)**
Adult	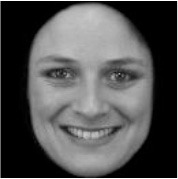	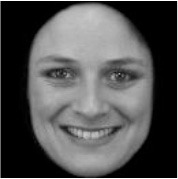	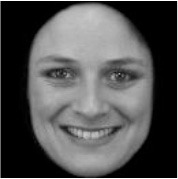
Infant	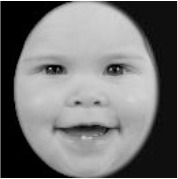	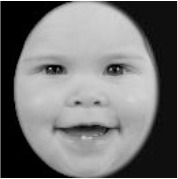	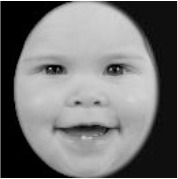

### Experimental Environment

MATLAB software (R2014b, MathWorks, MA, Psychtoolbox, 3) was used to display the experimental stimuli and record the responses of the participants. In a dark and sound-attenuated room, the visual stimuli were presented against a black background on a 24-inch VG 248 LCD (made by ASUS, Taiwan) computer monitor with a screen resolution of 1,920 × 1,080 and a refresh rate of 144 Hz. The distance between the computer monitor and the head of the participant was ~70 cm.

## Experiment 1: Paired Comparison Experiment

### Experimental Design

In this experiment, a pair of comparison methods was used to balance the influence of the model's own facial features on cuteness. This method allowed for more detailed comparisons. To avoid overwork, the experiment was divided into adult and infant groups according to the age of the models. In each age group, photos of each model were compared only with other 8 pictures of the infants in pairs, but all pairs from the photos of the models were disrupted and randomly presented.

Since the default probability of being asked to compare the same stimuli was 50%, no comparison between the same stimuli was performed. Using the 9 different stimuli of the same model as an example, the same two photos were not compared repeatedly, the number of comparisons was provided by equation (1).


(1) $$\frac{n^{*}\left(n-1\right)}{2}=\frac{9^{*}(9-1)}{2}=36$$

To avoid the preference of the participants for stimuli on a certain side, the left and right sides were switched for the 36 pairs of stimuli. In each group, participants completed 5 blocks (36 pairs ^*^ 2 sides × 5 models = 360 trials per block). Therefore, each stimulus was compared with the other eight stimuli of the same model, and each pair of comparisons appeared 10 times. After each block, the participants were given a 5–10-min break.

### Procedure

**Figure 2:**
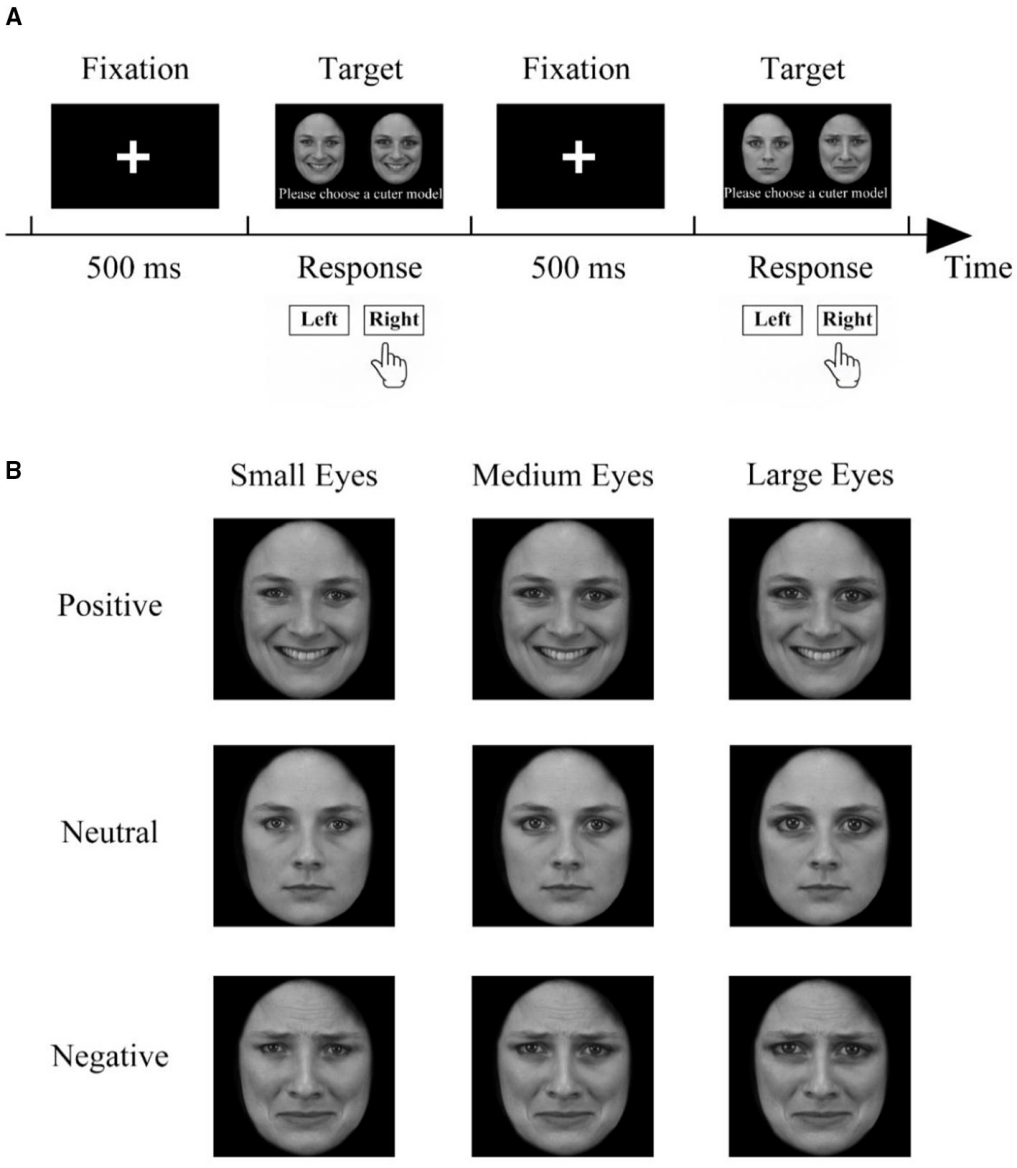
The paired-comparisons procedure. **(A)** The experimental process of a pair of comparative methods. First, the fixation point was displayed for 500 ms in the center of the screen, and then two stimuli of the same model with different features appeared at the same time. The participants were asked to choose the one they considered cuter. After the participant made a choice, a new set of fixed points was presented. **(B)** Example photos of the same model for pairwise comparison of 9 stimuli from the same model (no stimulus is compared with itself).

Figure 2 shows the procedure for the paired comparison experiment. Each trial began with a central fixation cross that was shown for 500 ms. The fixation was meant to draw the attention of the participants so they would not miss the subsequent stimuli. Then, a pair of faces was presented on the left and right, equidistant from the central fixation cross. The participants were asked to pay attention to the screen during the fixation and to provide a response by pressing one of two keys (“1” or “3”) to indicate which face was cuter. There was no need for a quick response. After their response, the next trial started.

### Data Analysis

Using the results of the choices of the participants, the probability that the same group of stimuli was selected in 10 comparisons (such as left-to-right swapping) was obtained. No comparison was made between the same stimuli, and the default probability of the same stimuli being selected was set at 50%. The average probability of such selection was calculated in each column unit. Taking the same participant's selection result of the same model stimulus as a unit, the data were standardized as a Z score, and finally, the absolute value of the minimum value in this set of numbers was added to each score to obtain a new Z score with a starting value of 0.

A 3 eye size _(large,unmanipulated,small)_ × 3 facial expressions _(negative,neutral,andpositive)_ repeated-measures ANOVA was performed, where eye size, and facial expressions were a within-subject factor, and the Z score for cuteness probability was the outcome variable. All statistical procedures were performed on a Windows platform. Studentized residuals (SREs) were calculated to verify that the data were in accordance with a normal distribution. Previous studies have shown that outlier data should be identified and removed when the absolute value of the SRE is ≥3 (Osborne and Overbay, 2004; James et al., 2013). In this way, data of one participant in the adult and infant groups were found to be outliers, so data of this participant were deleted. For all significant effects, *post-hoc* Bonferroni correction was applied to multiple comparisons. The effect size was estimated by the partial eta squared measure. When appropriate, critical values were adjusted using the Greenhouse-Geisser correction for the violation of the assumption of sphericity. The effect size was calculated by using the partial eta squared (η^2^).

### Results

**Figure 3:**
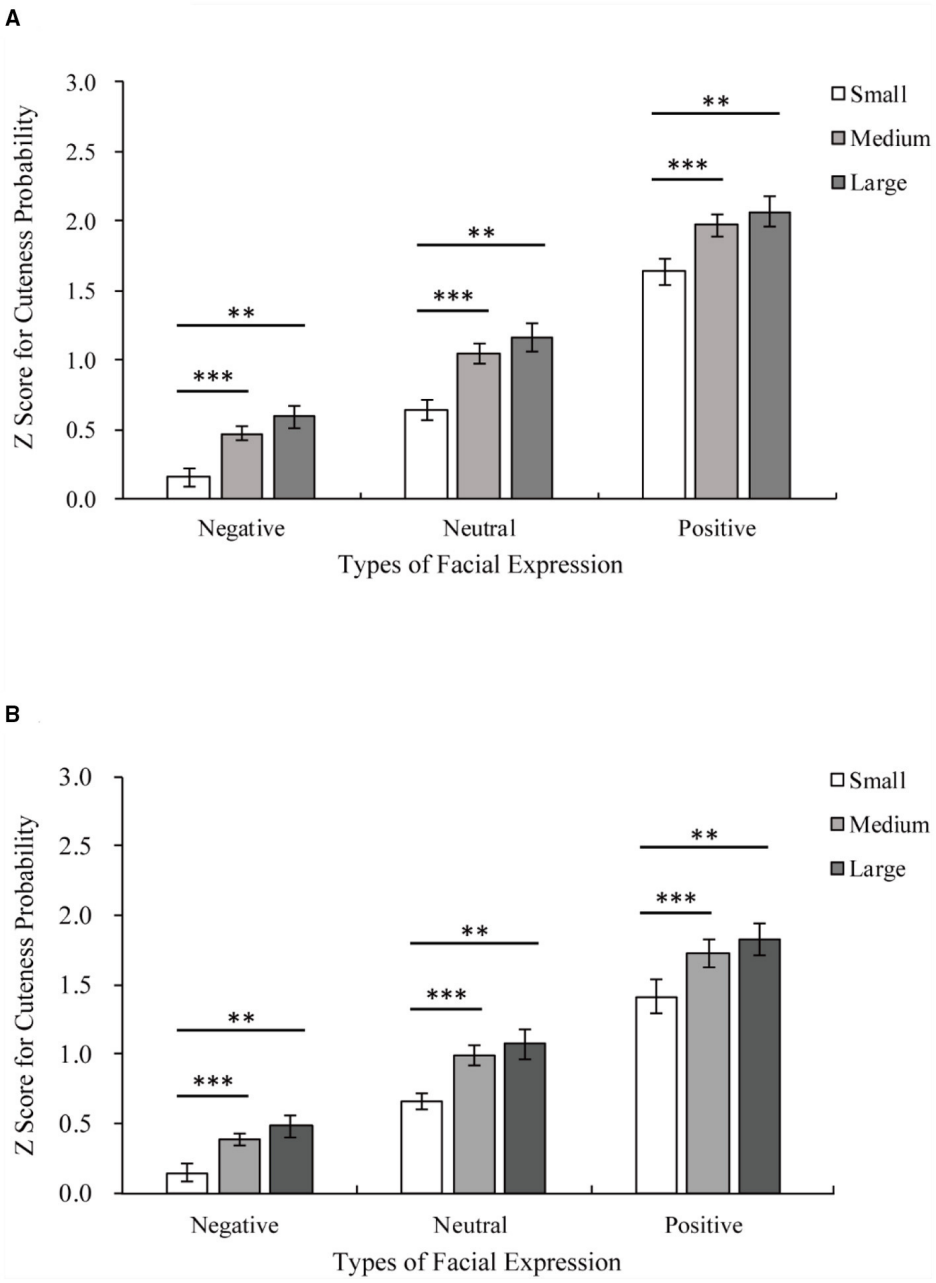
Z score for cuteness probability of adults and infants with the same facial expression. On the horizontal, axis are the three facial expressions of adults or infants, and on the vertical, axis are z scores for cuteness probability. **(A)** The results of the adult group. Medium eye > small eye among the same facial expression, all ***p < 0.001; large eye > small eye among the same facial expression, all **p < 0.01. **(B)** The results of the infant group. Medium eye > small eye among the same facial expression, all ***p < 0.001; large eye> small eye among the same facial expression, all **p < 0.01.

The results of the adult group are shown in Figure 3A. The main effects of facial expression [facial expression main effect *F*_(1.54,33.81)_ = 250.16, *p* < 0.001, η^2^ = 0.92] and eye size [eye size main effect *F*_(1.09,23.94)_ = 20.47, *p* < 0.001, η^2^ = 0.48] on the Z score for cuteness probability were significant. Pairwise comparisons employing Bonferroni corrections revealed that large eyes and unmanipulated eyes were rated as cuter than small eyes (large vs. small *p* < 0.001; unmanipulated vs. small *p* < 0.001; large vs. unmanipulated *p* = 0.094). Positive expressions were rated as cuter than neutral and negative expressions (all *p* < 0.001).

**Figure 4:**
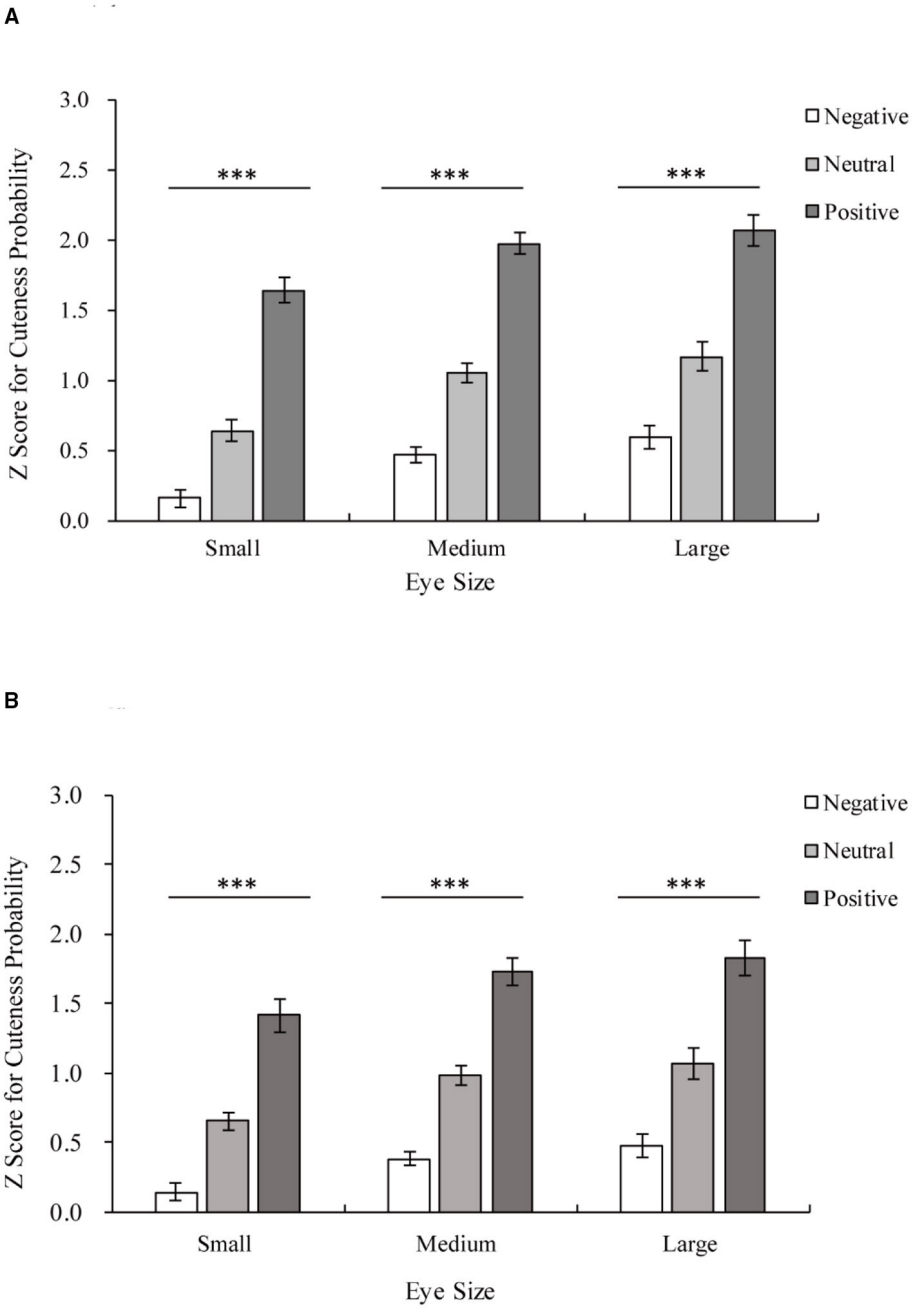
Z scores for the cuteness probability of adults and infants with the same eye size. The horizontal axis shows three levels of eye size, and the Z score for cuteness probability is shown on the vertical axis. White indicates negative stimuli, gray indicates neutral stimuli, and dark gray indicates positive stimuli. **(A)** The results of the adult group. Positive > Neutral > Negative among stimuli with the same eye size, all ***p < 0.001. **(B)** The results of the infant group. Positive > Neutral > Negative among stimuli with the same eye size, all ***p < 0.001.

Additionally, an interaction between facial expression and eye size was found [facial expression × eye size *F*_(4,88)_ = 4.79, *p* < 0.01, η^2^ = 0.18]. Positive expressions are rated as cuter than neutral and negative expressions for all eye sizes (all *p* < 0.001), as shown in Figure 4A. Both large eyes and unmanipulated eyes were rated as cuter than small eyes for all facial expressions (**Positive**: large vs. small *p* < 0.001; unmanipulated vs. small *p* < 0.001; large vs. unmanipulated *p* = 0.135; **Neutral**: large vs. small *p* < 0.001; unmanipulated vs. small *p* < 0.001; large vs. unmanipulated *p* = 0.159; **Negative**: large vs. small *p* < 0.001; unmanipulated vs. small *p* < 0.001; and large vs. unmanipulated *p* = 0.052).

The results for the **infant group** are shown in Figure 3B. There were significant main effects of facial expression [facial expression main effect *F*_(1.24,27.21)_ = 126.818, *p* < 0.001, η^2^ = 0.85] and eye size [eye size main effect *F*_(1.08,23.79)_ = 16.80, *p* < 0.001, η^2^ = 0.43] on the Z score for cuteness probability. Pairwise comparisons employing Bonferroni corrections revealed that large eyes and unmanipulated eyes were rated as cuter than small eyes (large vs. small *p* < 0.01; unmanipulated vs. small *p* < 0.001; large vs. unmanipulated *p* = 0.215). Positive expressions were rated as cuter than neutral and negative expressions (all *p* < 0.001). There is also an interaction between facial expression and eye size [facial expression × eye size *F*_(2.49,54.79)_ = 3.49, *p* < 0.05, η^2^ = 0.14], as shown in Figure 4B. Positive expressions were rated as cuter than neutral and negative expressions for all eye sizes (**all eye sizes:** positive vs. neutral *p* < 0.001; positive vs. negative *p* < 0.001; neutral vs. negative *p* < 0.001). Both large eyes and unmanipulated eyes were rated as cuter than small eyes for all facial expressions (**Positive:** large vs. small *p* < 0.01; unmanipulated vs. small *p* < 0.001; large vs. unmanipulated *p* = 0.20; **Neutral:** large vs. small *p* < 0.01; unmanipulated vs. small *p* < 0.001; large vs. unmanipulated *p* = 0.35; **Negative:** large vs. small *p* < 0.01; unmanipulated vs. small *p* < 0.001; and large vs. unmanipulated *p* = 0.18).

## Experiment 2: 7-Point Scale Experiment

### Experimental Design

In the cuteness evaluation task of experiment 2, all the stimuli were disordered and presented at random, and a 7-point scale was used to rate their cuteness. Since all stimuli were presented at random, the photos of different models could be compared with each other and could be used to investigate the effect of eye size on cuteness at different ages. Participants completed 5 blocks (90 trials per block).

### Procedure

**Figure 5:**
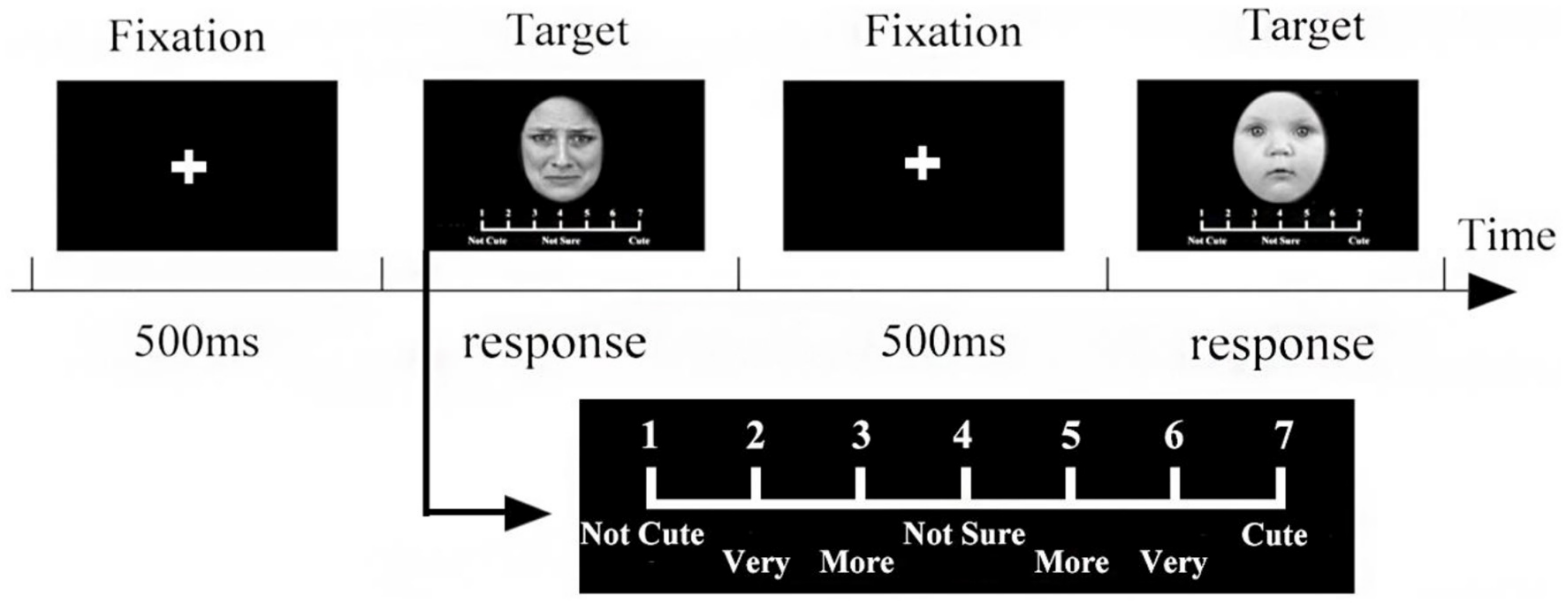
The 7-point scale procedure. After the fixation point was presented for 500 ms in the center of the screen, the stimuli were presented in a random order, and the evaluation scale was displayed under each stimulus. The participants were asked to evaluate the cuteness of each stimulus on a 7-point scale. After participants reached, the next fixed point was displayed in the center of the screen.

The procedure is shown in Figure 5. Each trial began with a central fixation cross that was shown for 500 ms. Then, 90 stimuli were randomly shown in the center (only one stimulus at a time). The participants rated the perceived cuteness on a scale of 1–7. After their response, the fixation point again appeared in the middle of the screen, prompting the participants to pay attention to it, and the next stimulus was then presented.

### Data Analysis

A 2 age (adults, infants) × 3 eye size (large, medium, small) × 3 facial expression (negative, neutral, and positive) repeated-measures ANOVA was performed, where age, eye size, and facial expression were within-subject factors and cuteness ratings were outcome variables. Similar to experiment 1, if the SREs were more than ±3, outliers were examined. It was found that the outliers in experiments 1 and 2 came from the same participant, so the data of this participant were also deleted in experiment 2. Normal distribution was not found in the three groups of photos of an infant with positive expressions divided by eye size. However, since the positive expressions of infants are likely to obtain a higher score than other expressions of infants, resulting in the irregular distribution of the data, the data were not converted and were analyzed. For all significant effects, *post-hoc* Bonferroni correction was applied for multiple comparisons. The effect size was estimated by the Partial Eta Squared measure. When appropriate, critical values were adjusted using the Greenhouse-Geisser correction for the violation of the assumption of sphericity. The effect size was calculated using the partial eta squared (η^2^).

### Results

**Figure 6:**
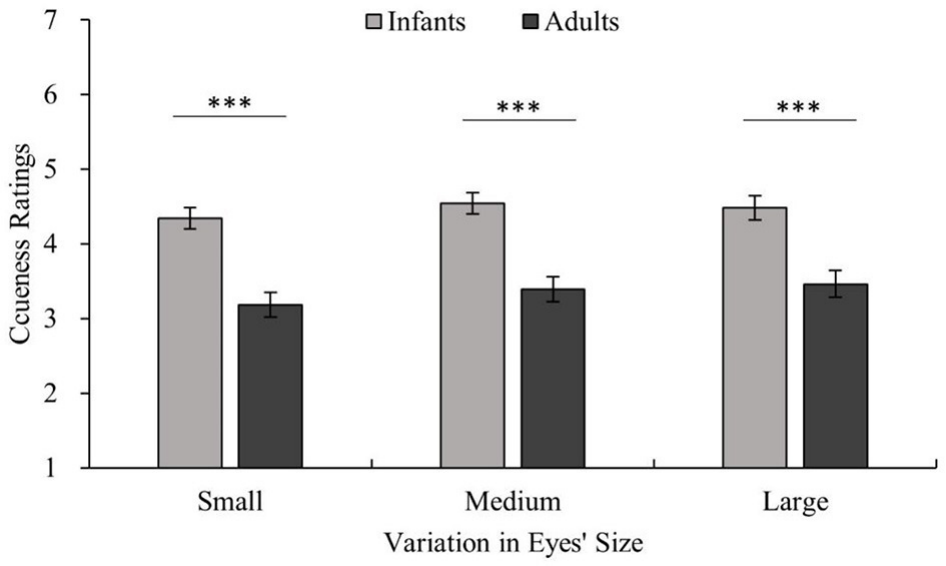
Ratings for the cuteness of infants and adults with the same eye size. The horizontal axis shows three eye sizes and cuteness ratings are shown on the vertical axis. The light color shows the infant stimuli, and the dark color shows the adult stimuli. Adults > infants for the same eye size, all ***p < 0.001.

**Figure 7:**
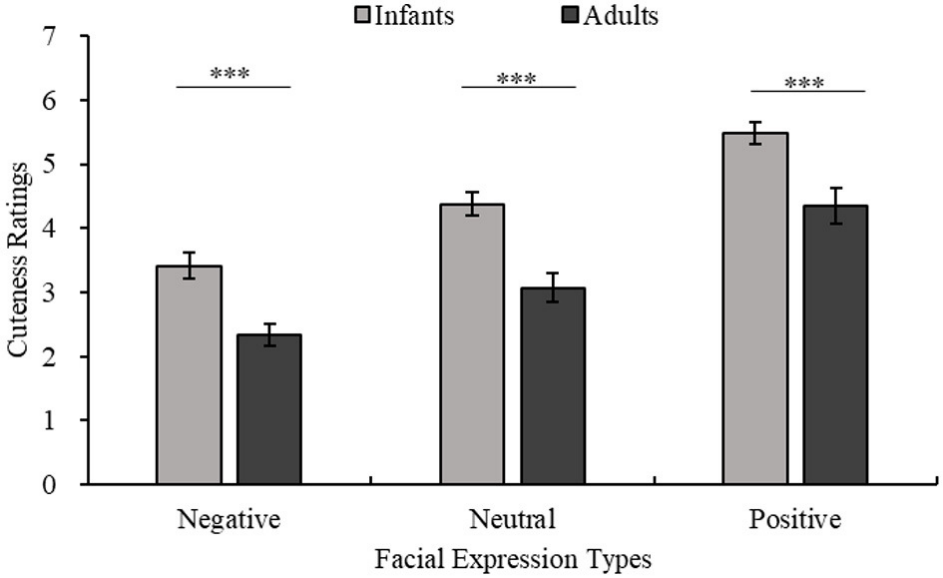
Ratings for the cuteness of infants and adults with the same facial expression. The horizontal axis shows three types of facial expressions, and cuteness ratings are shown on the vertical axis. The light color indicates the infant stimuli, and the dark color indicates the adult stimuli. Adults < infants with the same facial expression, all ***p < 0.001.

Since the main purpose of experiment 2 is to further examine the influence of age on the results of experiment 1, the age-related experimental results are marked in Figures 6, 7, and the remaining results are summarized as follows.

There were significant main effects of age [age main effect *F*_(1,22)_ = 22.02, *p* < 0.001, η^2^ = 0.50], facial expression [facial expression main effect *F*_(1.59,34.89)_ = 51.85, *p* < 0.001, η^2^ = 0.70], and eye size [eye size main effect *F*_(1.41,31.02)_ = 7.36, *p* < 0.01, η^2^ = 0.25] on cuteness ratings. Pairwise comparisons employing Bonferroni corrections revealed that the cuteness ratings of infants were higher than those of adults (infants vs. adults *p* < 0.001). Positive expressions were rated as cuter than neutral and negative expressions (positive vs. neutral *p* < 0.001; positive vs. negative *p* < 0.001; neutral vs. negative *p* < 0.001). Large eyes and unmanipulated eyes were rated as cuter than small eyes (large vs. small *p* < 0.05; unmanipulated vs. small *p* < 0.01; large vs. unmanipulated *p* = 1.00). Additionally, there was an interaction between facial expression and eye size that approached significance [facial expression × eye size *F*_(2.79,61.32)_ = 2.68, *p* = 0.059, η^2^ = 0.11]. However, there was no significant interaction between age and expression or age and eye size [age × facial expression × eye size *F*_(2.13,46.84)_ = 2.42, *p* = 0.097, η^2^ = 0.10].

## Discussion

In this study, the effect of eye size on perceived cuteness was investigated in which stimuli (adult and infant facial expressions) were objectively quantified according to eye size. The main finding of this study is that in both adults and infants, eye size has a significant effect on cuteness even in positive, neutral, and negative expressions. This shows that the influence of the baby schema on cuteness is more than just based on an overall change in all facial features. In the pairwise comparison method, stimuli involving the same model with different expressions and eye sizes were compared. Figure 3 shows that changing only eye size affects the perception of the participant of the cuteness of the model of the same age. This result supports the hypothesis: changing only the individual feature of eye size can affect cuteness perception. Based on the results of experiment 2, Figure 6 further shows that the change in cuteness caused by eye size does not make adults cuter than infants. This shows that even in different ages of stimuli, the hypothesis still holds. These results will be explained in terms of eye size, facial expression, and age below.

### Eye Size Effect and Cuteness

This study found that changing only the size of the eyes without changing other baby schema facial features can promote cuteness, as shown in Figure 3. As a unique and important facial feature, the eyes are good at attracting attention and expressing emotion. Compared to previous studies that have shown the effect of the baby schema on cuteness perception, this study focuses on the effect of an individual feature (eyes) and tests it in different facial expressions. The results show that changes in an individual feature, such as the eyes, in accordance with the baby schema can affect the perception of cuteness.

The results of the present study certainly show that changes in eye size objectively affect perceived cuteness. As stated above, cuteness is a protective mechanism for infants and children, and cuter facial features can attract attention and promote adult caretaking (Brosch and Sander, 2007; Glocker et al., 2009a,b; Luo et al., 2015b) so that children can be better protected. Woo et al. found that people focus more on the upper part of the face than on the lower part (Woo and Schaller, 2020). Some researchers have suggested that humans focus more attention on the eyes than on other facial features (Janik et al., 1978; Henderson et al., 2005). As mentioned in the Introduction, the results of Borgi et al's experiment in 2014 also somewhat support the view that humans usually focus on the eyes when looking at a face. Borgi et al. divide the face into three areas of interest (AOIs) corresponding to three key facial features: eyes, nose, and mouth. The demarcation method used in that study added 10 pixels to each facial feature, but the boundary between the nose and mouth was the midline between the upper lip and the bottom of the nose and was used to calculate the amount of attention each participant gave to different facial regions. The results show the eye area of the stimuli received the most viewing time (Borgi et al., 2014). When the eye size increases, that is, when a larger area is available for fixation, it is more likely to receive more attention from the observer, and cuter features become more perceptible. Alexandra Frischen also proposed that eyes and the surrounding region of the face can communicate complex mental states, such as emotions, beliefs, and desires; Frischen called these aspects the “language of the eyes” (Frischen, 2007). The eye also provides important social signals (Bar-On and Cohen, 1995; Taylor et al., 2001). In the study by Adolphs et al., it was found that patients with localized amygdala injury had difficulty judging scary expressions, a challenge that can be traced back to a lack of spontaneous eye gazing when freely viewing a face, thus making it difficult to properly use the information obtained from the eye area of the face (Adolphs et al., 2005). These results suggest that the eye is an important and unique facial feature in attracting the attention of others and expressing certain emotions. As mentioned previously, cuteness is a protective mechanism for infants that enables them to be better taken care of and can ensure their survival (Glocker et al., 2009a; Kringelbach et al., 2016). Larger eyes are in accord with baby schema; in other words, large eyes convey more infantile features and may cause observers to perceive a high level of cuteness. Therefore, the eyes may play an important role in the perception of observers of the cuteness of an object.

When perceiving facial cuteness, each facial feature may be analyzed independently. Kana Kuraguchi et al. found that the perception of the participants of cuteness was not affected by the face inversion effect (Kuraguchi and Kanari, 2020). Compared with upright faces, inverted faces are usually considered to interrupt second-order relational processing; in other words, they interrupt holistic processing (Hole et al., 1999). This suggests that the inability to holistically process the face does not affect the perception of cuteness (Kuraguchi and Kanari, 2020). In our study, changing the eye size also affected the facial ratio to some extent. However, based on the above research, we suggest that the effect of eye size on perceived cuteness is due mainly to eye size as an individual facial feature rather than to its proportional relationship to other facial features. These results indicate that the effect of facial feature changes on cuteness perception does not need to rely on overall features of baby schema and provides certain empirical support for studying the influence of single or local facial features on cuteness perception.

### Facial Expressions Effect and Cuteness

Aradhye et al. found that adults find smiling children cuter than children with neutral or crying expressions; thus, adults are more willing to adopt and take care of smiling children (Aradhye and Vonk, 2015). In the present study, Figure 4B shows that even with different eye sizes, infants with positive expressions are considered cuter than those with neutral or negative expressions. Additionally, as shown in Figure 4A, facial expressions of adults also influence their perceived cuteness. Related studies have shown that facial expressions can reflect emotions (Erickson and Schulkin, 2003), such as when babies smile at adults when playing with toys to convey their happiness (Cossette et al., 1996). At the same time, when external stimuli transmit positive emotions to the observer through positive expressions, the observer is more empathetic and positive (Kringelbach et al., 1994). This is due to the empathic ability of human beings in understanding other people's emotions and thoughts (Bellet and Maloney, 1991). Humans consider baby schema cute regardless of whether they appear in adults or children (Borgi et al., 2014). Many researchers believe that the unique feeling elicited by cute features should be classified as a positive emotion (Nittono et al., 2012; Buckley, 2016; Nittono, 2016; Laohakangvalvit et al., 2017). Moreover, this “cute-emotion” can encourage observers to socialize with specific objects by priming their affiliation and friendly tendencies (Nittono, 2016). Consistent with previous studies (Aradhye and Vonk, 2015), we also recognize that the positive or negative expressions of children convey positive or negative feelings to the observer. The results of the present study further suggest that this effect is not limited to children. Whether the object of observation is an adult or an infant, because of the effect of empathy, their positive or negative expressions seem to promote or inhibit the observer's positive emotions, such as the perceived cuteness.

Simultaneously, the participants perceived models with larger eyes to be cuter for models with positive, neutral, and negative expressions. This finding suggests that the effect of eye size on cuteness is not restricted by the overall facial expression. In previous research on facial perception, a common view is that the neural pathways of facial feature processing and face state processing differ (Haxby et al., 2000). The processing of facial features is the basis of individual perception, while the representation of facial status, for example, is the basis of social communication promoted by information perception (Haxby et al., 2000). A change in eye size is a change in facial traits, and different facial expressions represent different emotions conveyed by a face state (Pascalis et al., 2011). In the present study, although the interaction between expression and eyes was found using the *p*-value in the pairwise comparison experiment but the amount of each effect was too small (all <0.3), and the interaction was also not significant when the age factor was added in experiment 2. Thus, we also suggest that changes in the eyes and the observation of different expressions may not affect each other. Additionally, the observers judged the cuteness of a face, as shown in Figures 3, 4, both the expression of the stimuli and the eyes can influence the judgment of the observer. This finding shows that the nerve channels of facial feature processing and facial state processing transmit information regarding the cuteness of the face. This may speed up the processing of cuteness, which may be the reason why “cuteness” can be identified and responded to quickly. In other words, both facial expressions and eye sizes can affect observers' perception of cuteness, although they may be processed by different neural pathways. It could be speculated that changes in different facial expressions and the individual feature may affect cuteness perception in different ways.

### Aging Effect and Cuteness

Some researchers have found that when the object of observation has a neutral expression, both adult and child observers show a preference for the faces of babies over the faces of older children or adults (Luo and Li, 2011; Borgi et al., 2014; Luo et al., 2015a). In the present study, Figure 7 shows that children are still cuter than adults when conveying positive or negative expressions, confirming that the effect of facial expression on cuteness is not limited by age. Previous research shows that cuteness can promote human nurturing behavior, which is a potent protective mechanism of human beings that ensures the survival of otherwise completely dependent infants (Lorenz, 1943; Kringelbach et al., 2008, 2016; Glocker et al., 2009a,b). Studies have shown that the effect of baby schema seems to end when the child reaches four and a half years of age (Borgi et al., 2014). It is worth noting that at this time, the child is not yet developmentally close to being an adult. One view on this is that infant cuteness may decline with age because certain infantile features have weakened, but some features have probably not entirely disappeared even if the facial proportions of children at that age are close to those of adults (Luo and Li, 2011). “Cuteness” is a protective mechanism for the young (Kringelbach et al., 2016) and plays a diminishing role as children develop, which may make adults appear to be less cute than infants. Hence, we speculate that the decrease in children's cuteness may be related not to their growth and development toward a more adult form but to their certain individual infantile features becoming less obvious during development, which makes observers think that they no longer need to be protected. As mentioned above, the eyes are the part of the face that are most likely to be focused on. According to the results of the present study, smaller eyes are likely to be one of the features that make infantile features less obvious and tend to lead to a sense of adulthood. The results reaffirm the relationship between cuteness and infantile and support the previous view that certain crucial infantile facial cuts could make humans feel cuter (Luo and Li, 2011).

In this study, the participants without experience in taking care of infants were invited, as parenting experiences may make observers more sensitive to information about their infants. Therefore, the results of this study do not fully represent the responses of those with parenting experience. Given that cuteness and infantilization have a vital link, it is necessary to invite parents or people with parenting experience as participants to further explore primary communication mechanisms of the parent-child relationship in the future. Additionally, sex has not been considered in this paper. Future research should consider the effects of sex features to explore the effects of cuteness beyond the parenting instinct.

## Summary

Overall, eye size influences the perception of cuteness, even at different ages and with different facial expressions. Large eyes make the face seem cuter, even with different facial expressions or ages. Although eye size and expression type can influence the perception of cuteness, there was a weak correlation between the two in this study, possibly because of the different neural pathways along which the two features are transmitted. In addition, this study further verifies the finding of previous studies that facial expressions of the adults can affect their perceived cuteness. The result that infants are considered cuter than adults is not limited to faces with a neutral expression. In general, the results of this article verify and support that for humans without experience in taking care of infants, changing an individual facial feature in various facial expressions and ages has a moderating effect on their cuteness perception. This study still has certain limitations, which can be further researched in the future.

## Data Availability Statement

The original contributions presented in the study are included in the article/Supplementary Materials, further inquiries can be directed to the corresponding author/s.

## Ethics Statement

The studies involving human participants were reviewed and approved by the Ethics Committee of Okayama University. The patients/participants provided their written informed consent to participate in this study. Consent was obtained from owners of the database for the publication of any potentially identifiable images or data included in these researches.

## Author Contributions

LY, QD, YL, and QW conceived and designed the experiments. YY, TG, and MZ made the program. LY and QD collected the data, analyzed the data, then wrote the draft manuscript, and received comments from QW, YL, JY, ST, YE, and JW. All authors contributed to the article and approved the submitted version.

## Funding

This research was supported by the Japan Society for the Promotion of Science (JSPS) Kakenhi Grant Numbers 18H01411, 18K12149, 18K18835 19KK0099, 20K04381, 20K07722, and 21H05827; Shenzhen Overseas Innovation Team Project (No. KQTD20180413181834876); and a Grant-in-Aid for Strategic Research Promotion from Okayama University.

## Conflict of Interest

The authors declare that the research was conducted in the absence of any commercial or financial relationships that could be construed as a potential conflict of interest.

## Publisher's Note

All claims expressed in this article are solely those of the authors and do not necessarily represent those of their affiliated organizations, or those of the publisher, the editors and the reviewers. Any product that may be evaluated in this article, or claim that may be made by its manufacturer, is not guaranteed or endorsed by the publisher.
